# Computed Tomography Features of Parotid Tuberculosis

**DOI:** 10.1590/0037-8682-0455-2023

**Published:** 2023-10-30

**Authors:** Larissa Nobre Lopes de Lima, Fernando Carlos Santos de Almeida, Diogo Goulart Corrêa

**Affiliations:** 1 Universidade do Estado do Rio de Janeiro, Departamento de Medicina Interna, Disciplina de Radiologia, Rio de Janeiro, RJ, Brasil.; 2 Clínica de Diagnóstico por Imagem, Departamento de Radiologia, Rio de Janeiro, RJ, Brasil.

**FIGURE 1:**
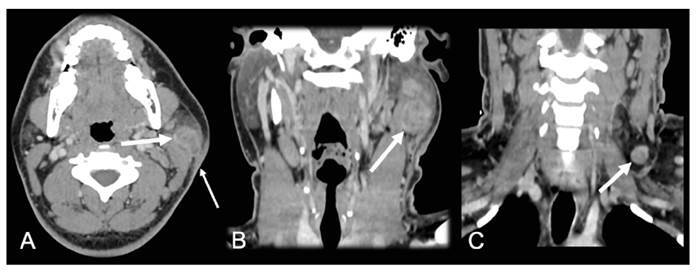
Primary parotid tuberculosis. Axial **(A)** and coronal **(B)** neck CT images showing an expansive lesion with heterogeneous enhancement and a central area of necrosis in the superficial lobe of the left parotid gland **(thick arrow in A, arrow in B)**. The main parotid duct had a normal caliber. Sialolithiasis was absent. Also note the skin fistulization of the lesion **(thin arrow in A)**. Cervical lymph node enlargement was present ipsilateral to the lesion, in the coronal plane **(arrow in C)**.

A 27-year-old man presented with enlargement of the left cervical region, fever, and weight loss for the past month. Physical examination revealed left cervical lymph node enlargement with skin fistulization. Hemogram and chest computed tomography (CT) findings were normal. Neck CT revealed a mass with heterogeneous contrast enhancement and central necrosis in the left parotid, with fistulization to the skin and lymph node enlargement (Figure 1). Analysis of a fine-needle aspiration sample yielded a positive GeneXpert^®^ MTB/RIF assay result, suggesting tuberculosis. Serological analysis was negative for human immunodeficiency virus. Culture was positive for *Mycobacterium tuberculosis*. The patient was treated with rifampicin, isoniazid, pyrazinamide, and ethambutol.

Tuberculosis rarely involves the salivary glands because antibacterial activity and continuous saliva flow prevent the accumulation of the bacillus. Among the salivary glands, the parotid glands are most commonly involved because of their slower saliva flow^1^. A primary pulmonary focus and hematogenous and/or lymphatic dissemination usually characterizes parotid involvement but dissemination via the parotid duct from an oral-cavity or lymph-node focus can also occur^2^. CT usually shows a lobulated heterogeneously enhancing mass, associated with lymph-node enlargement. Primary neoplasms and other infectious diseases, such as other bacterial and fungal infections, are important differential diagnoses^1^ ^,^ ^3^. In the Brazilian public health system, the GeneXpert^®^ MTB/RIF assay is an important tool for diagnosing tuberculosis. It detects *M. tuberculosis* genetic material and rifampicin resistance within two hours^4^. Therefore, tuberculosis should be considered in cases presenting with parotid lesions to avoid unnecessary treatment and procedures.
